# Histone Deacetylase 2 Knockdown Ameliorates Morphological Abnormalities of Dendritic Branches and Spines to Improve Synaptic Plasticity in an APP/PS1 Transgenic Mouse Model

**DOI:** 10.3389/fnmol.2021.782375

**Published:** 2021-11-24

**Authors:** Daiki Nakatsuka, Takaya Izumi, Tasuku Tsukamoto, Miki Oyama, Kohei Nishitomi, Yuichi Deguchi, Kazuki Niidome, Hidekuni Yamakawa, Hisanori Ito, Koichi Ogawa

**Affiliations:** Shionogi & Co., Ltd., Osaka, Japan

**Keywords:** HDAC2, Alzheimer’s disease, dendritic morphology, double transgenic mice, amyloid precursor protein, learning and memory

## Abstract

Disease-modifying therapies, such as neuroprotective and neurorestorative interventions, are strongly desired for Alzheimer’s disease (AD) treatment. Several studies have suggested that histone deacetylase 2 (HDAC2) inhibition can exhibit disease-modifying effects in AD patients. However, whether HDAC2 inhibition shows neuroprotective and neurorestorative effects under neuropathic conditions, such as amyloid β (Aβ)-elevated states, remains poorly understood. Here, we performed HDAC2-specific knockdown in CA1 pyramidal cells and showed that HDAC2 knockdown increased the length of dendrites and the number of mushroom-like spines of CA1 basal dendrites in APP/PS1 transgenic mouse model. Furthermore, HDAC2 knockdown also ameliorated the deficits in hippocampal CA1 long-term potentiation and memory impairment in contextual fear conditioning tests. Taken together, our results support the notion that specific inhibition of HDAC2 has the potential to slow the disease progression of AD through ameliorating Aβ-induced neuronal impairments.

## Introduction

Alzheimer’s disease (AD) is a progressive neurodegenerative disorder characterized by the deposition of amyloid β(Aβ) plaques and accumulation of intracellular neurofibrillary tangles, which is followed by degenerative morphological changes and neuronal dysfunction (Paulson et al., 2008). Specifically, dendritic length and numbers of dendritic branching and spines are reduced in hippocampal neurons of AD patients (Adlard and Vickers, 2002; Falke et al., 2003; Grutzendler et al., 2007) and AD mouse models, including amyloid precursor protein (APP) transgenic mice such as presenilin1 (PS1) and APP double transgenic mice (Grutzendler et al., 2007; Malthankar-Phatak et al., 2012; Šišková et al., 2014). Until now, there have been no approved therapies for AD with proven neuroprotective and neurorestorative effects, termed disease-modifying effects, which are defined to affect the disease process by decreasing neuronal death and slowing down the clinical course (Cummings, 2009). Therefore, it is important to identify molecules related to the disease-modifying effect and develop new therapies to stop disease progression.

Epigenetics, including histone acetylation, have gained particular attention for their role in several learning and memory processes (Alarcón et al., 2004; Korzus et al., 2004; Taniguchi et al., 2017). Non-selective histone deacetylase (HDAC) inhibitors restore spine impairment and memory function (Fischer et al., 2007; Ricobaraza et al., 2012), suggesting that HDAC inhibitors are one of the candidates to develop AD therapies that could have disease-modifying effects. Non-selective HDAC inhibition also causes several undesirable effects, such as hematological and neurological toxicity (Gojo et al., 2007; Marks and Xu, 2009; Prince et al., 2009). Therefore, non-selective HDAC inhibition may cause serious adverse effects in patients with AD.

Recently, HDAC2, a member of the HDAC family, has been identified as a regulator of structural and functional plasticity in the nervous system. HDAC2 levels are elevated in both AD patients and AD mouse models (Gräff et al., 2012; Gonzalez-Zuñiga et al., 2014). Furthermore, it has been shown that knockdown of HDAC2 improves memory impairment in neurodegenerative model mice (Guan et al., 2009; Gräff et al., 2012). Moreover, HDAC2-specific inhibition induces dendritic and spine growth both *in vivo* and *in vitro* in wild-type mice (Nott et al., 2008; Guan et al., 2009). Notably, loss of HDAC2 does not affect the number of splenocytes, thymocytes, or bone marrow cells, suggesting that the specific inhibition of HDAC2 reduces the risk of side effects (Yamaguchi et al., 2010; Heideman et al., 2014). Taken together, HDAC2-specific inhibition may be a suitable therapy for AD.

However, it is not clear whether specific inhibition of HDAC2 affects disease progression, such as impairment of dendrite and spine morphology in the neuropathic state, such as amyloid plaque elevation. In this study, to investigate whether HDAC2-specific inhibition has potential disease-modifying effects in the neuropathic state, we examined the effect of HDAC2 knockdown on neuronal morphology, synaptic plasticity, and memory function in doubly transgenic mice (PS/APP) overexpressing mutant APP and PS1 transgenes.

## Materials and Methods

### Animals

Double transgenic mice (Tg-APP/PS1, referred to as PS/APP mice, male) were generated by crossing the well-established APP mutant line, Tg2576, purchased from Taconic Biosciences (Leverkusen, Germany), with heterozygous PSEN1^I213T^ KI mice [named PS mice; (Nakano et al., 1999)] gifted by Department of Psychiatry, Osaka University Medical School. PS mice, non-transgenic littermate mice (wild-type mice), and C57BL/6J Jcl mice (B6 mice, purchased from CLEA Japan, Inc.) were used as controls. The mice were housed in a temperature- (23 ± 2°C) and humidity-controlled (50 ± 10%) environment under a 12 -h light/dark cycle (lights on at 8:00). The mice were housed 1–5 mice per cage. All experiments were approved by the Animal Care and Use Committee of the Shionogi Research Laboratories.

### Experimental Manipulations of Histone Deacetylase 2

To suppress gene expression of HDAC2, we used adeno-associated virus serotype 9 (AAV9)-CAG-microRNA (miR)-HDAC2 targeting the following sequence: 5′- TGCTGTAACATAGCAGAACC CTGATGGTTTTGGCCAC TGACTGACCATCAGGGCTGCTATGTTA-3′. The AAV9-CAG-miR-Ctrl targeting control sequence (5′-TGCTGA AATGTACTGCGCGTGGAGACGTTTTGGCCACTG ACTGA CGTCTCCACGCAGTACATTT-3′) was used as a control vector. These AAV vectors (titers: 2 × 10^13^ genome copies/ml, volume: 1 μl) were bilaterally injected into the CA1 region of the hippocampus (from bregma, AP:-2.0, ML: ± 1.6, DV:1.7 mm) using a stereotaxic apparatus.

### Measurement of Aβ

To investigate Aβ accumulation in PS/APP mice, brain samples were collected from 4- to 12-month old PS/APP, PS, and wild-type mice (5 mice for each group). The hemisphere of each brain was homogenized in 1% Triton X-100 in Tris-buffered saline (20 mM Tris and 137 mM NaCl, pH 7.6) with Complete™ protease inhibitor (Roche Diagnostics, Indianapolis, IN, United States). Brain homogenates were ultracentrifuged to separate 1% Triton X-100-soluble from -insoluble fractions at 70000 rpm for 20 min at 4°C. The insoluble proteins were sonicated using 5 M guanidine in Tris-buffered saline with Complete™ protease inhibitor and then centrifuged at 14000 rpm for 20 min at 4°C. All samples were diluted at least 1:10. Aβ40 and Aβ42 levels were determined using a sandwich ELISA kit (Biosource International, Camarillo, CA, United States). To investigate the Aβ reduction by HDAC2 knockdown, brain samples were collected from 7-month-old mice at 3 months after AAV injection (4 mice for each group). Mice that underwent the fear conditioning test were reused. Unilateral hippocampal samples including CA1 region was homogenized in 1% SDS in 50 mM Tris-HCL buffer with Complete™ protease inhibitor (Roche Diagnostics, Indianapolis, IN, United States) and PhosSTOP (Roche Diagnostics, Indianapolis, IN, United States). The brain homogenates were ultracentrifuged at 16000 g for 15 min at 4°C and Aβ40 and Aβ42 levels in the supernatant were determined using human βAmyloid (1–40) ELISA and βAmyloid (1–42) ELISA kits (Fujifilm Wako Pure Chemical, Osaka, Japan), respectively.

### Measurement of Histone Deacetylase mRNA and Protein

To investigate the efficacy of AAV-mediated HDAC2 silencing, we performed quantitative PCR (qPCR) and western blotting. Hippocampal samples from the bilateral CA1 region were collected from 6-to 7-month-old mice (6–8 mice from each group), 7–8 weeks after AAV injection. The half amount of hippocampal sample was used for qPCR experiments and the remaining half of hippocampal sample were used for western blotting experiments. For qPCR experiments, RNA was extracted using RNeasy Mini Kit (Qiagen, West Sussex, United Kingdom). 150 ng RNA was reverse-transcribed using High-Capacity RNA-to-cDNA™ Kit (Applied Biosystems, Waltham, MA, United States). qPCR was carried out using SYBR Premix ExTaq™ II (TAKARA Biotech, Japan). Relative mRNA expression was calculated using the ΔΔCT method by using GAPDH as an internal control. The primers used for qPCR experiments were as follows: Mouse Hdac2 forward: 5′-ACCACATGCACCTGGTGTTCA-3′; Mouse Hdac2 reverse: 5′-TCGCAAGCTATCCGTTTGTCTG-3′; Mouse GAPDH forward: 5′- TGTGTCCGTCGTGGATCTGA -3′; Mouse GAPDH reverse: 5′- TTGCTGTTGAAGTCGCAGGAG -3′. For western blotting experiments, protein was extracted from the hippocampal samples (4 mice from each group) and 10 μg of total proteins for each sample was quantified using BCA protein assay. The proteins were transferred onto PVDF membrane and blot was placed into blocking solution for 1 h at room temperature. The blot was incubated with Anti-HDAC2 antibody (#ab12169, 1:10000, Abcam, Cambridge, MA, United States) at 4°C for overnight. Anti-rabbit HRP-linked Antibody (#7074, 1:1000, Cell Signaling Technology) was added and incubated for 1 h at room temperature. The protein bands were visualized by ECL methods and captured with LAS3000 (Fujifilm, Tokyo, Japan). β-Actin (13E5) Rabbit mAb (#4970, 1:1000, Cell Signaling Technology, Dancers, United States) was used as the internal standard. The western blots were quantified using ImageJ.

### Golgi Staining Procedure

To investigate the aberration in neuronal morphology of PS/APP mice, brain samples were collected from 8-month-old mice. To assess the effects of HDAC2 knockdown, brain samples were collected from 7-month-old mice at 3 months after AAV injection. For the HDAC2 knockdown experiment, mice that underwent the fear conditioning test were reused. Golgi staining was performed using the FD Rapid GolgiStain kit (FD NeuroTechnologies, Ellicott City, MD, United States). The hemisphere of each brain were immersed in a solution of equal parts Solution A and Solution B for 2 weeks, and then soaked in Solution C for 1 week. After freezing with isopentane chilled with dry ice, the brain samples were sliced into 100 μm pieces using Leica CM 1850 and mounted on glass slides. Slides were stained with a mixture (Solution D: Solution E: DW; 1:1:2) and protected with Permount reagent (Fisher Scientific). Bright field images were obtained using a BZ-9000 digital microscope (Keyence Corporation, Osaka, Japan). Dendrites and spines were analyzed using ImageJ software (National Institutes of Health). For the dendritic analysis, basal dendrites of CA1 pyramidal neurons were selected. For the spine analysis, terminal dendrites which located more than 40 μm from the soma and have a length more than 20 μm were selected. Mushroom spines were defined as having a large head of the spine with a neck. The experimenter was analyzed under blind conditions. In the experiment for aberration in neuronal morphology, 31–42 neurons from 3 mice for each group (B6: 12, 9, 10, PS: 11, 13, 15, PS/APP: 16, 15, 11 neurons per mouse) were used for dendritic analysis, and 12–17 terminal dendrites from 3 mice for each group (B6: 3, 4, 5, PS: 6, 5, 6, PS/APP: 2, 7, 3 neurons per mouse) were used for spine analysis. In the HDAC2 knockdown experiment, 36–49 neurons from 4 mice for each group (RNAi-Ctrl: 10, 14, 16, 9, RNAi-HDAC2: 10, 8, 11, 7 neurons per mouse) were used for dendritic analysis, and 14–21 terminal dendrites from 4 mice for each group (RNAi-Ctrl: 6, 7, 6, 2, RNAi-HADC2: 4, 3, 3, 4 neurons per mouse) were used for spine analysis.

### Slice Preparation and Electrophysiology

Brain samples were collected from 6-to 7-month-old mice. To assess the effects of HDAC2 knockdown, brain samples were collected 1 month after AAV injection. Transverse hippocampal slices (400 μm thick) were prepared using a vibratome (VT1200S; Leica, Germany). Field excitatory postsynaptic potentials (fEPSPs) were evoked by stimulation in Schaffer collaterals and recorded in the CA1 area with MED64 (Alpha MED Scientific, Inc, Japan). After recording a stable baseline for 15 min, long-term potentiation (LTP) was induced by theta burst stimulation (TBS) consisting of 10 trains of 4 pulses at 100 Hz with an inter-train interval of 200 ms. The stimulus intensity was set to 30–50% of the maximum response. LTP recording was performed 150 min after the TBS. The slices exhibiting unstable baseline recordings were discarded. 8–13 slices from 3–5 mice for each group (2.6 ± 0.2 slices from each mouse) were analyzed for the LTP abnormality experiment of PS/APP mice. 19 slices from 7–9 mice for each group (2.4 ± 0.3 slices from each mouse) were analyzed for the HDAC2 knockdown experiment.

### Contextual Fear Conditioning

As described in Figure 3D, 1 month after AAV injection, 6-to 7-month old mice (16–25 mice from each group) were placed in the conditioning chamber and were exposed to three electric foot shocks (0.2 mA, 2 s duration, and 80 s apart). After the last shock, mice were kept in the chamber for another 10 s and returned to their home cages. On the testing day (24 h after conditioning), the animals were placed back into the conditioning chamber for 5 min. All experiments were analyzed using an automatic video tracking system (TimeFZ2 software; O’Hara & Co., Ltd.) to compare frame-by-frame movements to determine the amount of time spent freezing. Immobility was defined as a movement of less than 20 pixels for a training session or 30 pixels for a test session.

**FIGURE 1:**
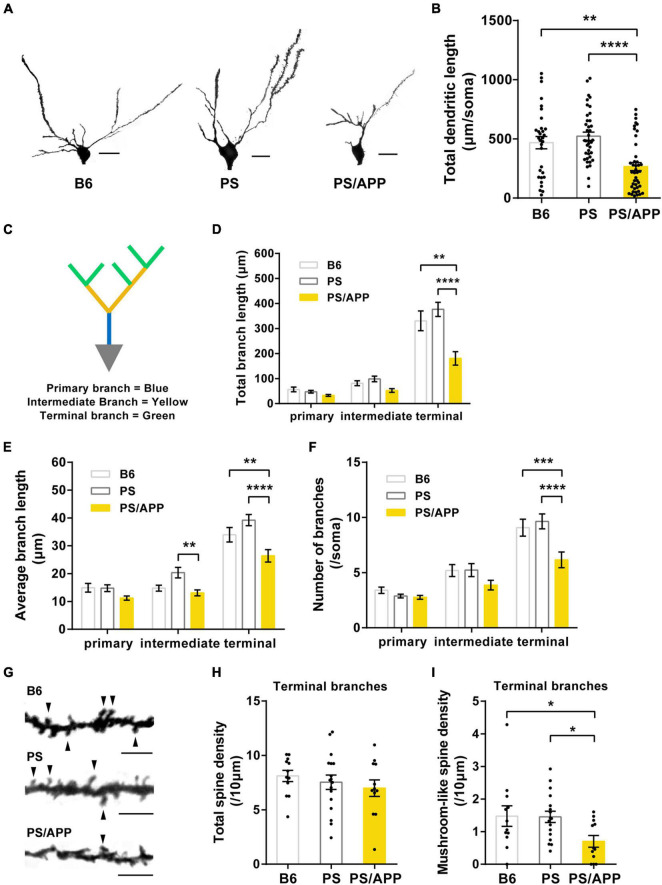
Dendrites and mushroom-like spines were impaired in PS/APP mice. **(A)** Extracted representative Golgi staining images of CA1 basal dendrites. The scale bar is 20 μm. **(B)** Total length of CA1 basal dendrite. In PS/APP mice, total length was significantly decreased compared with B6 and PS mice. **(C)** An image of a CA1 pyramidal cell depicting the breakdown of the arbor into branch types. **(D)** Total length of each order of dendrite. PS/APP mice showed a decrease in total length of terminal branches. **(E)** Average length of each dendritic order. In PS/APP mice, length of intermediate and terminal branches was significantly decreased. **(F)** Number of branches at each order of dendrite. PS/APP mice showed significant decrease in number of terminal branches. **(G)** Extracted representative images of spine on basal dendrite. Arrowheads indicate mushroom spines. The scale bar is 5 μm. **(H)** Density of total spine on terminal branches. There were no significant changes between all groups. **(I)** Density of mushroom spines in terminal branches. PS/APP mice showed significant decrease in mushroom-like spines. All data are presented as mean ± SEM. *p < 0.05, **p < 0.01, ***p < 0.001, and ****p < 0.0001. Dunn’s multiple comparisons test for **(B)**, two-way ANOVA, followed by Dunnett’s multiple comparisons test for **(D–F)**, Dunnett’s multiple comparisons test for **(H,I)**.

**FIGURE 2:**
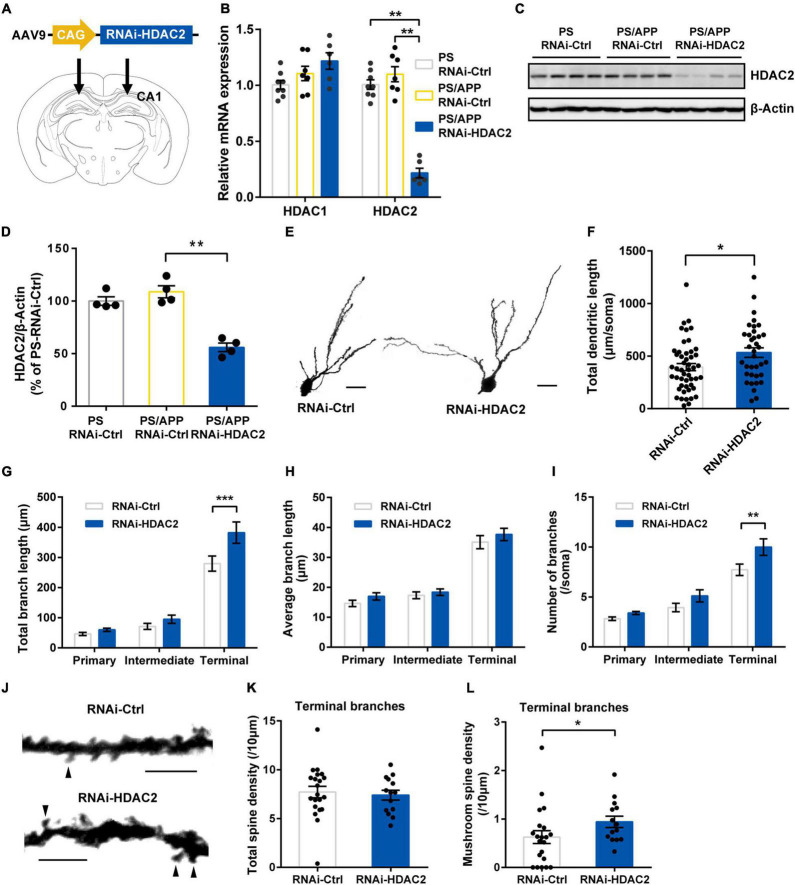
Histone Deacetylase 2 silencing ameliorated morphological impairment in PS/APP mice. **(A)** Schematic representation of AAV-RNAi-HDAC2 and injection site. **(B)** Relative mRNA expression of HDAC1 and HDAC2 in the hippocampal CA1 region. HDAC2 mRNA level was decreased in PS/APP mice injected with AAV-RNAi-HDAC2. **(C)** Western blot analysis of HDAC2 proteins. Each band represents an individual mouse. HDAC2 protein level was decreased in PS/APP mice injected with AAV-RNAi-HDAC2. **(D)** Quantified western blots of HDAC2. AAV-RNAi-HDAC2 significantly suppressed HDAC2 protein expression. **(E)** Extracted representative images of CA1 basal dendrite. The scale bar is 20 μm. **(F)** Total length of CA1 basal dendrites. HDAC2 silencing significantly increased total dendritic length in PS/APP mice. **(G)** Total length of each order of dendrite. HDAC2 silencing increased length of terminal branches. **(H)** Average length of each order of dendrite. There was no significant difference. **(I)** Number of branches at each order of dendrite. HDAC2 silencing increased number of branches of terminal dendrites. **(J)** Extracted representative pictures of spines on terminal branches. Arrowheads indicate mushroom spines. The scale bar is 5 μm. **(K)** Density of total spines in terminal branches of CA2 basal dendrite. There was no significant change. **(L)** Density of mushroom-like spines in terminal branches of CA1 basal dendrite. HDAC2 silencing significantly increased mushroom-like spines. All data are presented as mean ± SEM. *p < 0.05, **p < 0.01, and ***p < 0.001. Dunn’s multiple comparisons test for **(B,D)**, Mann Whitney test for **(F,K,L)**. Two-way repeated measure ANOVA, followed by Sidak’s multiple comparisons test for **(G–I)**.

**FIGURE 3:**
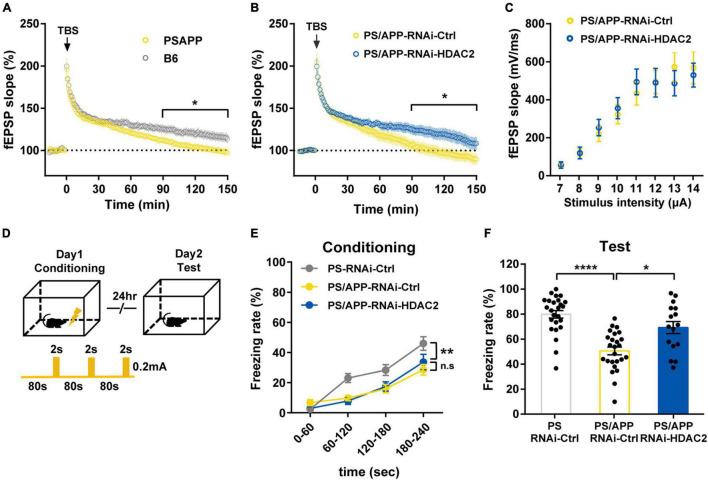
Histone Deacetylase 2 silencing ameliorated the impairment of synaptic plasticity and memory in PS/APP mice. **(A)** LTP from PS/APP mice decayed to the baseline. **(B)** LTP induced by 4 trains of TBS. HDAC2 silencing improved the decay observed in PS/APP mice. **(C)** Input/output relationships from PS/APP mice injected with AAV-RNAi-HDAC2 or AAV-RNAi-Ctrl. There was no significant difference. **(D)** Schematic of the experimental procedure of fear conditioning test. **(E)** Freezing rate at conditioning trial. PS mice and PS/APP mice showed progressive increase of freezing behavior. **(F)** Freezing rate at test trial. PS/APP mice showed decrease in freezing behavior. HDAC2 silencing improved the decreased freezing behavior of PS/APP mice. All data are presented as mean ± SEM. *p < 0.05, **p < 0.01, and ****p < 0.0001. Two-way repeated measured ANOVA for **(A,B,E)**, two-way ANOVA for **(C)**, Dunn’s multiple comparisons test for **(F)**.

### Statistical Analysis

Statistical analyses were performed using the GraphPad Prism version 6. For two-group comparisons, the Mann-Whitney test were used. For three-group comparisons, Dunnett’s multiple comparisons test or Dunn’s multiple comparisons test were used depending on the distribution. For repeated measures, two-way repeated measures ANOVA was performed. All data are presented as the mean ± SEM. Statistical significance was set at *p* < 0.05.

## Results

### Impairment of Dendritic Morphology and Spines in CA1 Basal Dendrite of PS/APP Mice

It has been reported that the dendritic length, branch, and density of mushroom-like spines in basal dendrites were decreased in hippocampal CA1 pyramidal neurons of AD model mice (Perez-Cruz et al., 2011; Šišková et al., 2014). To investigate the aberration in neuronal morphology of PS/APP mice, we confirmed that the insoluble Aβ increased from 4 months in the brain of PS/APP mice (Supplementary Figure 1). We then analyzed Golgi-stained pyramidal neurons in the CA1 basal region of 8-month-old mice (Figure 1A). The total length of CA1 basal dendrites in PS/APP mice was significantly lower than that in PS and B6 mice (Figure 1B). Given that different dendritic regions assume different roles (Bloss et al., 2016), we performed a detailed morphometric analysis to distinguish different dendritic regions. Following precedents from previous research (Bloss et al., 2016), we separated the dendritic regions into primary, intermediate, and terminal branches (Figure 1C). Interestingly, the total length was significantly reduced, especially in the terminal branches of PS/APP mice compared with PS or B6 mice (Figure 1D). In terminal branches, the average length and number of branches decreased in PS/APP mice (Figures 1E,F). These results indicate that the terminal branches are more vulnerable than the primary and intermediate dendrites in PS/APP mice. Next, to evaluate spine abnormality, we counted the number of spines on the terminal branches (Figure 1G). There was no difference in the total spine density of terminal branches among B6, PS, and PS/APP mice (Figure 1H). However, the density of mushroom-like spines was significantly reduced in PS/APP mice compared with that in PS or B6 mice (Figure 1I). Collectively, these findings suggest that the terminal branches are especially impaired in PS/APP mice showing Aβ accumulation, compared with PS or B6 mice.

### Histone Deacetylase 2 Knockdown Ameliorated Dendrite Deficits and Spine Abnormality in PS/APP Mice

Histone deacetylase 2 inhibition is known to induce dendritic and spine growth under naïve conditions (Nott et al., 2008; Guan et al., 2009). To examine whether specific inhibition of HDAC2 ameliorates deficits in neuronal morphology under Aβ- accumulation, we injected adeno-associated virus carrying microRNA targeting HDAC2 (RNAi-HDAC2) or control sequence (RNAi-Ctrl) into the CA1 region of PS/APP mice (Figure 2A). We confirmed that HDAC1 mRNA levels were not significantly different between RNAi-HDAC2 and RNAi-Ctrl groups, while HDAC2 mRNA levels of PS/APP mice injected with RNAi-HDAC2 (PS/APP-HDAC2-RNAi) group was decreased to 19.7% in PS/APP mice injected with RNAi-Ctrl (PS/APP-RNAi-Ctrl) and significantly reduced when compared with both PS mice injected with RNAi-Ctrl (PS-RNAi-Ctrl) and PS/APP-RNAi-Ctrl groups (Figure 2B). In addition, HDAC2 protein levels also significantly decreased (Figures 2C,D). These results indicate that the gene knockdown of HDAC2 by RNAi-HDAC2 is effective and allows for examination of the effect of HDAC2 knockdown in PS/APP mice. HDAC2 knockdown did not changed Aβ levels in CA1 region (Supplementary Figure 2). Next, to assess whether HDAC2 gene knockdown improves neural impairment in Aβ accumulated condition, Golgi analyses were performed on the CA1 basal region of PS/APP-RNAi-HDAC2 (Figure 2E). HDAC2 knockdown significantly increased total dendritic length in PS/APP mice (Figure 2F). An increase in total dendritic length was detected, especially in terminal branches (Figure 2G). Although HDAC2 knockdown did not affect the average length for every order of dendrites (Figure 2H), the numbers of terminal branches were significantly increased by HDAC2 knockdown (Figure 2I). Furthermore, we examined whether HDAC2 knockdown affected spine morphology in terminal dendrites (Figure 2J). HDAC2 knockdown did not change total spine density (Figure 2K), although mushroom-like spine density was significantly increased in PS/APP-RNAi-HDAC2 (Figure 2L). These results suggest that specific HDAC2 inhibition can ameliorate dendritic impairments, especially on terminal branches, even under Aβ-accumulated conditions.

### Histone Deacetylase 2 Knockdown Ameliorated the Impairment of Long-Term Potentiation and Episodic Memory in PS/APP Mice

Since dendritic and spine geometry is inseparably linked with neural function (Hausser et al., 2000; Poirazi and Mel, 2001; Spruston, 2008), to examine whether HDAC2 knockdown improves synaptic plasticity, we performed recordings of field excitatory postsynaptic potentials (fEPSPs) in the CA1 of PS/APP mice for long-term potentiation (LTP) analysis. LTP induced by theta burst stimulation (TBS) rapidly decayed in 6-to 7-month-old PS/APP mice compared with B6 mice (Figure 3A), indicating synaptic plasticity deficits in PS/APP mice. On the other hand, PS/APP-RNAi-HDAC2 maintained the late phase of LTP compared with the PS/APP-RNAi-Ctrl group (Figure 3B). The input-output relationship was not different between the PS/APP-RNAi-HDAC2 and PS/APP-RNAi-Ctrl groups (Figure 3C). These results indicate that HDAC2 knockdown ameliorates the impairment of synaptic plasticity without effect on the efficiency of synaptic transmission and/or neural excitability in PS/APP mice. In addition, to verify the functional consequence of the improvement of synaptic plasticity, we tested the effect of HDAC2 knockdown on memory function in 6-to 7-month-old PS/APP mice using a contextual fear conditioning test (Figure 3D). In the conditioning trial (Figure 3E), both PS and PS/APP mice showed a progressive increase in freezing behavior following foot shock. The freezing rate of PS/APP mice was lower than that of PS mice, although there was no significant difference between the PS/APP-RNAi-HDAC2 and PS/APP-RNAi-Ctrl groups. Twenty-four h after the conditioning session, mice were placed back into the conditioning chamber, and freezing behavior was scored without foot shock (Figure 3F). The PS/APP-RNAi-Ctrl group showed decreased freezing behavior compared with the PS-RNAi-Ctrl group. In contrast, the PS/APP-RNAi-HDAC2 group exhibited increased freezing behavior than the PS/APP-RNAi-Ctrl group and at similar levels to PS mice-RNAi-Ctrl. These findings suggest that HDAC2 inhibition improves synaptic plasticity and episodic memory in PS/APP mice showing Aβ accumulation.

## Discussion

Although several studies have reported the role of HDAC2 in learning and memory (Nott et al., 2008; Guan et al., 2009; Gräff et al., 2012), whether HDAC2 inhibition has disease-modifying effects in the neuropathic state, such as in Aβ-induced conditions, is still unknown. To shed light on this question, we performed knockdown of HDAC2 in PS/APP mice using AAV encoding miRNA of HDAC2, and discovered that HDAC2 inhibition improved dendritic morphologies, synaptic plasticity, and episodic memory even under Aβ elevated conditions (Supplementary Figure 3).

### Histone Deacetylase 2 Knockdown Ameliorates Disruption of Neuronal Morphology and Function Under Neuropathic Condition

In the neuropathic state, such as amyloid plaque-elevated conditions of AD, whether inhibition of HDAC2 affects the disease process, such as impairment of dendrite and spine geometry is still unknown. Here, under Aβ elevated conditions, we examined the effects of HDAC2 knockdown and found that HDAC2 knockdown ameliorated dendritic impairments and increased postsynaptic mushroom-like spines. Given that HDAC2 inhibition also increases presynaptic vesicle protein synaptophysin in another AD model mice, neurodegenerative model mice (Gräff et al., 2012), the effects of HDAC2 inhibition are thought to cover a wide range of neuronal morphology in several neuropathic states. In addition, we observed that HDAC2 knockdown increased LTP, as previously reported in neurodegenerative model mice (Gräff et al., 2012). In this study, enhancement of LTP was observed, especially in the late phase of LTP. Since late-phase LTP is known to require new protein synthesis and gene transcription (Lynch, 2004), and HDAC inhibitors have been reported to enhance synaptic plasticity and memory function through cAMP response element binding protein (CREB)-dependent transcriptional activation (Vecsey et al., 2007), the benefits of HDAC2 knockdown are suggested to be induced by transcriptional activation. Taken together, these findings suggest that HDAC2 inhibition can maintain neuronal morphology and function *via* transcriptional activation in several neuropathic states.

### Amelioration of Neural Circuits Involved in Learning and Memory by Histone Deacetylase 2 Specific Inhibition

Several studies have shown that synaptic spines and dendrites are impaired in the basal dendrites of the CA1 region in APP overexpression mice (Perez-Cruz et al., 2011; Šišková et al., 2014). Our results revealed that dendrites in PS/APP mice were impaired, especially in terminal branches, and HDAC2 knockdown rescued the branching of terminal dendrite. Since brain derived neurotrophic factor (BDNF) is a well-established HDAC2 target gene, BDNF signaling can be enhanced by HDAC2 inhibition (Penney and Tsai, 2014). And BDNF signaling is also known to increase dendritic branching (Horch and Katz, 2002). In addition, dendrites are known to have homeostatic function such as dendritic self-avoidance to avoid overlapping with other dendrites (Grueber and Sagasti, 2010), suggesting that the part of impaired dendrites can be more recoverable. Therefore, enhanced BDNF signaling by HDAC2 knockdown can contribute to increase the number of branches especially in terminal dendrites which are most vulnerable to Aβ accumulation. Although PS/APP mice exhibited the impairments of both the branch length and the number of branches, HDAC2 knockdown did not ameliorate the branch length but the number of branches. However, as for the branch length, we assumed that the effect of HDAC2 knockdown was not detectable rather than that HDAC2 knockdown was not able to ameliorate, because the impairment of the branch length was weaker than the impairment of the number of branches in the HDAC2 knockdown experiments.

In addition to the effects on neuronal morphology, HDAC2 knockdown also improved episodic memory. From these results, it could be hypothesized that improvement of basal dendrites underlies the amelioration of memory deficit. This hypothesis does not contradict previous studies that reported that an increase in spines and dendrites is induced by learning and prevention of cognition, specifically in the basal dendrites of CA1 pyramidal cells (Leuner et al., 2003; Vila-Luna et al., 2012). CA1 basal dendrites are known to receive both excitatory inputs from CA2 pyramidal neurons and inhibitory inputs from bistratified cells (Klausberger and Somogyi, 2008; Kohara et al., 2014). A recent electrophysiological study showed that recruitment of CA2 pyramidal neurons into the CA3-CA1 circuit increases the excitatory drive between CA3 and CA1, which is thought to be involved in memory formation (Nasrallah et al., 2019). In addition, inhibitory inputs such as CA1 bistratified cells have been reported as an instrumental for the theta phased activation of hippocampal neurons, which is known to be important for the encoding and retrieval of memories (Cutsuridis and Poirazi, 2015). Notably, the majority of excitatory synapses and 66% of inhibitory synapses are known to contact the terminal branches of CA1 basal dendrites (Bloss et al., 2016), suggesting that terminal dendrites are key regions for hippocampal memory circuits. Altogether, these results support the hypothesis that the amelioration of memory deficit by HDAC2 knockdown is, at least partially, attributed to the morphological improvement of CA1 basal dendrites, although we cannot exclude the possibility that some alteration on CA1 apical dendrites induced by HDAC2 knockdown is also involved in memory improvement. Furthermore, although the contextual fear conditioning test is the behavioral paradigm used to assess associative fear learning and memory in rodents (LeDoux, 2000), fear conditioning test alone is not sufficient to completely understand memory function. Therefore, to understand a whole picture of the mechanism of memory improvement by HDAC2 inhibition, other memory tests such as Morris water maze are required to evaluate other memory function such as spatial memory.

### Histone Deacetylase 2 Specific Inhibition Has the Potential to Be a Disease-Modifying Therapy Even Under Aβ-Elevated Conditions

In this study, our findings demonstrate that HDAC2 knockdown ameliorates neural impairment followed by improvement of synaptic and memory function in PS/APP mice.

Current drugs for AD, such as acetylcholinesterase inhibitors and N-methyl-d-aspartate (NMDA) antagonists, are symptomatic treatments that only ameliorate the symptoms (Citron, 2010). Therefore, disease-modifying therapies, such as interventions with neuroprotective or neurorestorative effects, are strongly desired (Cummings, 2009). The potential of non-selective HDAC inhibition for disease-modifying effects has recently been suggested using glycogen synthase kinase 3β (GSK-3β)/HDAC dual inhibitor (De Simone et al., 2019). In this study, it was confirmed that HDAC2 silencing improves neuronal morphology and memory function in PS/APP mice, confirming that HDAC2 inhibition could be a disease-modifying therapy. Aβ plaques, neurofibrillary tangles and neuronal cell death are pathological hallmarks of AD (Paulson et al., 2008). Class I and II HDAC inhibitors decrease Aβ expression and HDAC2 silencing reduces Aβ-induced neuronal death (Qing et al., 2008; Ricobaraza et al., 2011; Sung et al., 2013; Wang et al., 2019). However, in our experiments, HDAC2 knockdown was not able to reduce Aβ accumulation at least in our experimental condition. Therefore, the neuroprotective effect such as reducing Aβ accumulation is not supposed to be the main factor to ameliorate neuronal morphology in our study. However, in order to conclude this, we need more comprehensive evaluation for other neuropathic factors such as tau pathology which has been shown to be inhibited by Class I and II HDAC inhibition (Ricobaraza et al., 2009). In addition, neuronal cell death is also important hallmark of Alzheimer’s disease. However, we have not evaluated neuronal loss in this study because according to the previous study which demonstrated neuronal loss of CA1 neuron is not induced at 12-month-old PS1 and APP double transgenic mice (Takeuchi et al., 2000). We assumed that neuronal cell death is not induced in 6 to 8 months old of our PS/APP mice which we used in this study. Taken together, in our study, we assume that the effects of HDAC2 knockdown on neuronal morphology and memory functions was induced without effecting to amyloid plaque formation and neuronal cell death. On the other hand, as mentioned above, our results suggested that HDAC2 knockdown has neurorestorative effects *via* transcriptional activation. Thus, HDAC2 inhibition might show disease-modifying effects through mainly neurorestorative effects. However, although disease-modifying therapy is strictly defined by slowing down disease progression and needs to be demonstrated by long-term experiments (Cummings, 2009), our study has limitations because we examined the effect of HDAC2 knockdown only at one time point. Therefore, in order to confirm that HDAC2 inhibition has a disease-modifying effect, it is necessary to investigate the effects of HDAC2 inhibition over long observation periods.

## Data Availability Statement

The raw data supporting the conclusions of this article will be made available by the authors, without undue reservation.

## Ethics Statement

The animal study was reviewed and approved by Animal Care and Use Committee of the Shionogi Research Laboratories.

## Author Contributions

DN generated and analyzed the data and drafted the manuscript. TI, TT, MO, and KoN generated the data. YD, KaN, HY, and KO provided the study concept and design. HI and KO supervised the study. All authors read and approved the final manuscript.

## Conflict of Interest

All authors were employed by Shionogi & Co., Ltd. at the time of data collection and manuscript writing. This study received funding from Shionogi & Co., Ltd. The funder had the following involvement with the study: the decision to submit it for publication.

## Publisher’s Note

All claims expressed in this article are solely those of the authors and do not necessarily represent those of their affiliated organizations, or those of the publisher, the editors and the reviewers. Any product that may be evaluated in this article, or claim that may be made by its manufacturer, is not guaranteed or endorsed by the publisher.
