# Supplementary material for: Histone Deacetylase 2 Knockdown Ameliorates Morphological Abnormalities of Dendritic Branches and Spines to Improve Synaptic Plasticity in an APP/PS1 Transgenic Mouse Model
Source: Front Mol Neurosci. 2021 Nov 24;14:782375. doi: 10.3389/fnmol.2021.782375 (PMC8652290; doi:10.3389/fnmol.2021.782375)
Supplement: Supplementary file 1 [file Data_Sheet_1.docx]

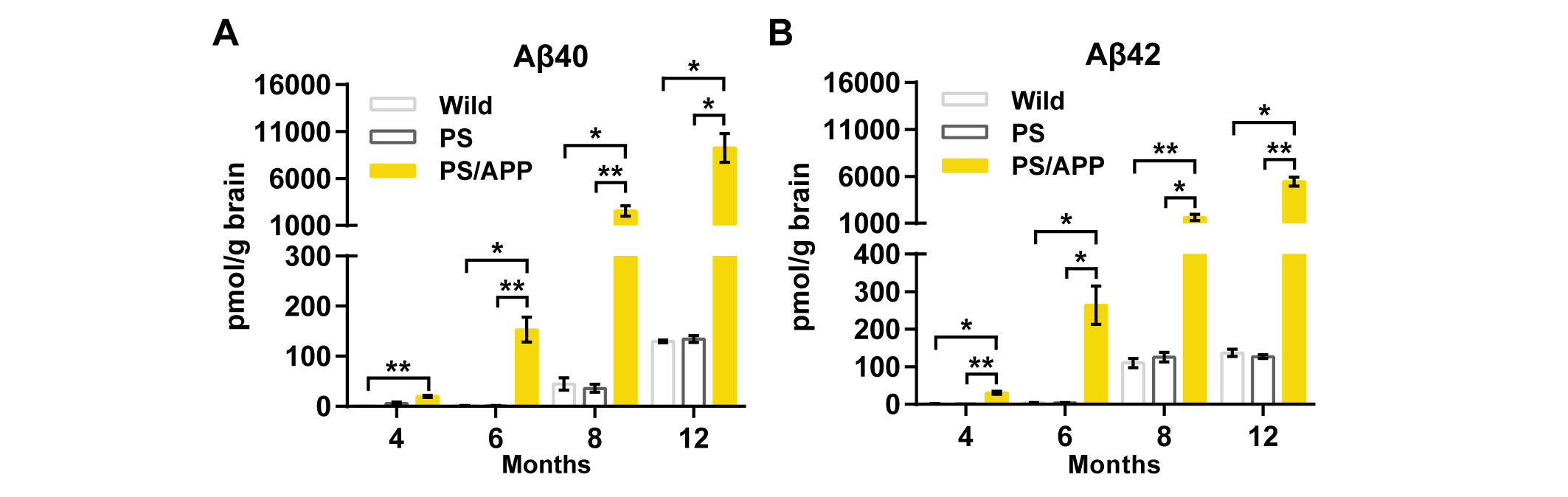


**Supplementary Figure 1.** Aβ accumulation in PS/APP mice. (A) Insoluble brain Aβ40. (B) Insoluble brain Aβ42. From 4 months, insoluble Aβ40/42 was accumulated in PS/APP mice. Values are presented as mean ± SEM. ^∗^p < 0.05 and ^∗∗^p < 0.01, Dunn's multiple comparisons test.


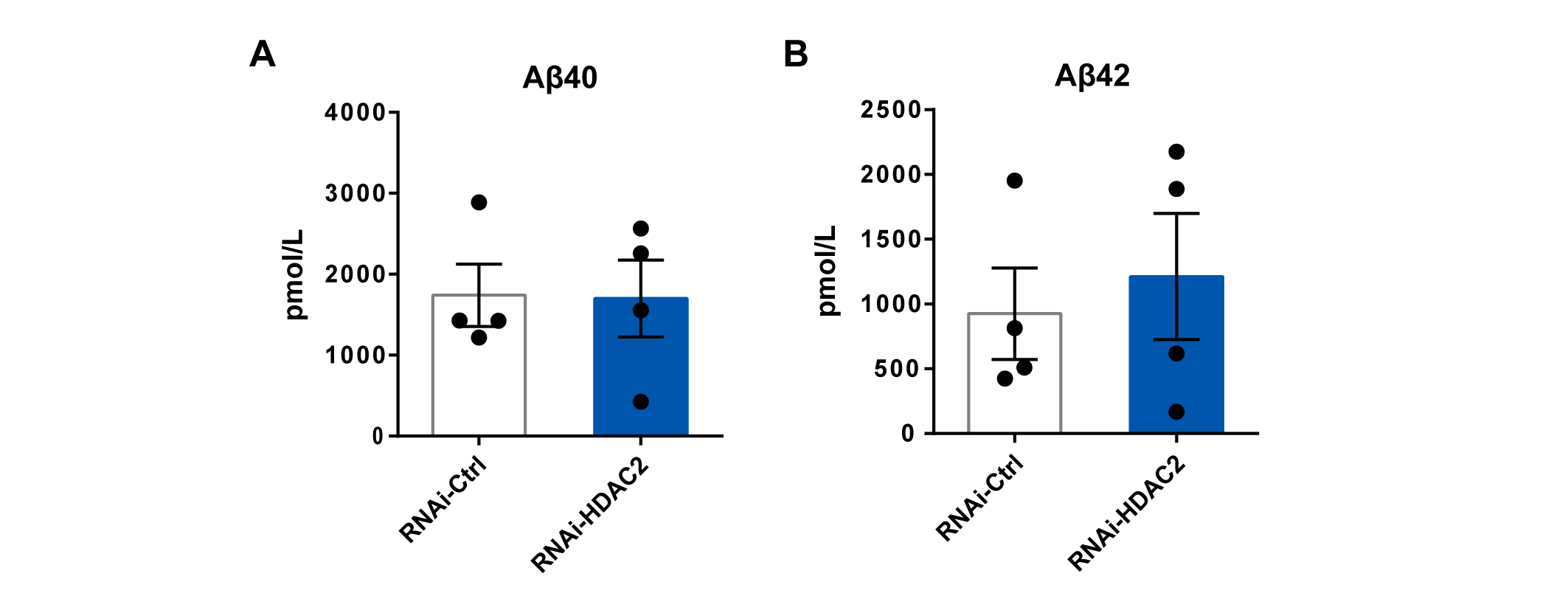


**Supplementary Figure 2.** The effects of HDAC2 knockdown on Aβ levels in the CA1 of PS/APP mice. (A) Aβ40 and (B) Aβ42 in CA1 region. HDAC2 knockdown tended to reduce Aβ level in CA1 region, but not significant. Values are presented as mean ± SEM.


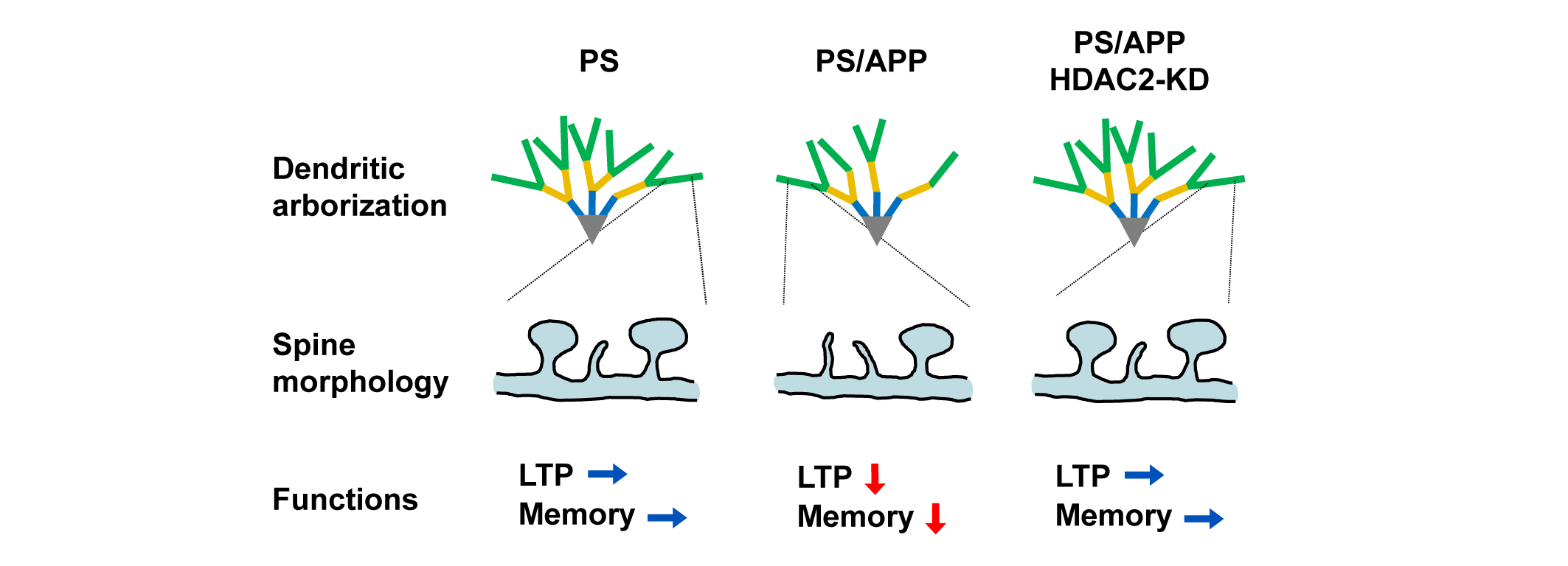


**Supplementary Figure 3**. Graphical abstract for the effects of HDAC2 knockdown on neuronal morphology and memory functions. HDAC2 knockdown ameliorated dendritic morphologies, synaptic plasticity, and episodic memory even under Aβ elevated conditions.
